# Histologic changes in systemically loaded right ventricles at time of transplantation

**DOI:** 10.3389/fcvm.2025.1640561

**Published:** 2025-10-20

**Authors:** Riya Nilkant, Perry S. Choi, Danielle M. Mullis, Robin Chang, Amit Sharir, Sushma Reddy, Glenn J. Pelletier, Michael Ma

**Affiliations:** ^1^ Department of Cardiothoracic Surgery, Stanford University, Stanford, CA, United States; ^2^ Department of Pediatrics (Cardiology), Stanford University, Stanford, CA, United States

**Keywords:** systemic right ventricle, ventricular failure, heart transplantation, histology, ventricular tissue, fibrosis

## Abstract

**Introduction:**

In a subset of complex congenital heart disease patients, the right ventricle (RV) is connected to the higher-resistance systemic circulation, often leading to RV dysfunction. This study characterizes the systemic RV histology at time of heart transplantation in two groups — patients with hypoplastic left heart syndrome with Fontan palliation (HLHS-F) and those with dextro-transposition of the great arteries post atrial switch operation (d-TGA-AS). We sought to better understand histological differences between the systemic RV in the single ventricle vs. biventricular circulations.

**Methods:**

We procured RV tissue samples at the mid-cavity free walls from nine explanted recipient hearts: six HLHS-F and three d-TGA-AS. RV and LV muscle samples from two organ donors whose hearts were unused for non-cardiac reasons served as controls. Tissue sections were stained with Masson's Trichrome and Hematoxylin and Eosin. Given the small cohort size and heterogeneity, analyses were descriptive. Continuous variables are reported as median (range).

**Results:**

The d-TGA-AS population was older than the HLHS-F population (median: 42 years; range: 38–44 vs. median: 24 years; range: 12–32, respectively). RV fibrosis in the d-TGA-AS population was greater at 28% (7–35) vs. the HLHS-F population which was 4% (2–24) and donor controls (median: 2% range: 0–4). In contrast, RV wall thickness was greater in HLHS-F (median: 12,303 µm; range: 9,976–15,745) than in d-TGA-AS (median: 9,063 µm; range: 8,316–10,322) and donors (median 7,984 µm; range: 2,582–13,386). Donor LV thickness (median: 17,056 µm; range: 16,688–17,423) exceeded all RV groups.

**Conclusion:**

The primary histologic finding for the d-TGA-AS group was fibrosis, while the HLHS-F group showed predominantly hypertrophy. The temporal presentation of the patients was different, with the HLHS-F patients presenting earlier for transplant than the d-TGA-AS. These observations suggest that different histologic changes may occur in response to longstanding systemic pressures in these two anatomic subgroups of patients with systemic RV.

## 1 Introduction

Congenital heart disease (CHD) remains the most common birth defect and a leading cause of infant death. In a subgroup of these patients, the right ventricle (RV) which normally pumps to the low-pressure pulmonary circulation instead pumps to the higher-pressure systemic circulation. These patients are at particular risk for arrhythmias, RV enlargement and dysfunction, and tricuspid regurgitation (1).

These patients can further be divided into single and biventricular subgroups. For some types of single ventricle CHD, such as Hypoplastic Left Heart Syndrome (HLHS), the RV initially serves as the primary pump for both the pulmonary and systemic circulations in parallel, but following staged palliation it becomes exclusively the systemic pump. In contrast, there are congenital heart malformations in a biventricular system in which the RV pumps to the systemic circulation and the LV pumps to the pulmonary circulation, such as in dextro-transposition of the great arteries (d-TGA) following an atrial switch operation (d-TGA-AS).

The palliative atrial switch was designed to divert systemic venous blood from the right atrium to the left ventricle, with pulmonary venous blood directed to the right ventricle. Thus, cyanosis is corrected, but the RV remains in the systemic circulation, and the LV pumps to the pulmonary arteries. The current gold standard for surgical repair of d-TGA is the arterial switch, however, older patients who had the atrial switch operation still exist. Despite initial success with surgical palliation, long-term outcomes demonstrate that only 68% of d-TGA-AS patients remain alive in the fourth decade of life, and their leading cause of hospitalization is heart failure (2, 3). With the growing number of patients with d-TGA-AS presenting with deterioration of the systemic RV, the need for further investigation becomes prudent (4).

In both patients with HLHS with Fontan palliation (HLHS-F) and d-TGA-AS, the systemic RV physiology has been shown to ultimately lead to RV systolic failure (1). Fifty percent of d-TGA-AS patients present with heart failure in the 4th to 5th decade of life; however, no effective medical therapy exists (5, 6).

Thus, treatment of systemic right ventricular failure remains a challenge. While the failure of the systemic left ventricle has been extensively studied, the mechanisms by which the right ventricle fails are less understood. It has been shown that RV failure is different from that of the LV (7). Furthermore, therapies that benefit patients with systemic LV failure have not produced similar results in patients with systemic right ventricular failure (6, 7). Heart transplant is a definitive treatment for heart failure but given that transplant waiting list time could be long, developing more effective therapies for systemic RV failure is imperative.

This study aims to characterize the histological differences of the systemic right ventricle at the time of transplant in single ventricular and biventricular circulations. To achieve this aim, we collected right ventricular muscle from the excised hearts of patients with systemic RV at the time of transplantation. Through histological staining, we characterized myocardial fibrosis and thickness and compared them to tissue collected from donor hearts unused for noncardiac reasons. These results not only unveil potential contributors to systemic RV failure, but also help elucidate histological differences between failed systemic RV in patients with HLHS–F and those with d-TGA-AS. Such new information may better inform therapies that could reduce the need for transplantation.

## 2 Methods

We harvested ventricular muscle samples from the explanted hearts of nine transplant recipients at the time of transplant according to our Institutional Review Board protocols #55285, #80565, and #32769. All 9 patients had systemic RV physiology: 6 in the HLHS-F group and 3 in the d-TGA-AS group. RV and LV muscle samples from donor hearts (48-year-old male and 53-year-old male) not used for noncardiac reasons served as controls. Indications for transplant were systemic right ventricular failure in both groups.

Upon removal of the recipient heart, we immediately placed the explanted organ on ice. We excised a full-thickness piece of ventricular muscle from each explanted heart at the midpoint of the RV free wall and from a similar site in the control RV and LV. These sections were then filled with Fisher Tissue Plus Optimal Cutting Temperature Compound (#4585, Waltham, MA), immediately frozen using dry ice/methylbutane, and stored at −80°C. We cut serial cryosections (10 μm) using an Epredia Cryostar NX90 cryostat (Thermo Fischer, Fisher Scientific, USA) and placed them onto charged slides. All samples were kept at −80°C until stained.

We used StatLab's Masson's Trichrome Stain Kit (StatLab, Cat: KTMTR EA, McKinney, TX) to stain collagen fibers in each sample. Collagen and fibrous tissue stained blue, cytoplasm stained red, and nuclei stained black. To measure the thickness of the ventricle and cell architecture we stained each sample with hematoxylin and eosin (H&E) using the Thermo Scientific Shandon Rapid-Chrome H&E Frozen Section Staining Kit (Thermo Fisher Scientific, Cat: 9990001).

We imaged the stained tissue samples using a Keyence BZ-X810 microscope (Keyence, Osaka, Japan) and Plan Apochromato 10× BZ-PA10 and Plan Apochromato 4× BZ-PA4 lenses. For the samples stained with Masson's trichrome, nine 10× images were taken of all ventricular samples, which included three each from epicardium, myocardium, and endocardium. For the H&E staining, we obtained one 4× image of the entire tissue sample.

Image analyses were quantified using Keyence BZ-X800 Analysis software (Keyence, Osaka, Japan). For the Masson's Trichrome staining, we calculated the percentage of fibrosis, by dividing the area of fibrosis (blue) by the total area of tissue for each sample (blue and red). For the H&E staining, we measured the thickness of the ventricular sample—the epicardium to endocardium length—under 4× power.

Given the small cohort and heterogeneity, all analyses were descriptive. Continuous variables were summarized as median (range) and categorical variables as counts with percentages. No formal hypothesis tests or confidence intervals were performed. Technical replicates within each region were collapsed by median, and regional medians were then collapsed to generate a single representative value for the right or left ventricle of that patient. Group-level results are reported as number of patients (n), median, and range (min–max) of these per-patient ventricular values. Tables display individual values where available to ensure transparency.

## 3 Results

### 3.1 Demographics

Demographic and clinical characteristics for each patient are listed in Table 1 and summarized in Table 2. Six HLHS-F patients and three d-TGA-AS patients were included. The HLHS-F group had three females compared to one female in the d-TGA-AS group. The HLHS-F group tended to be younger (median: 24 years; range: 12–32) compared to the d-TGA-AS group (median: 42 years; range: 38–44). Median waitlist time was longer for HLHS-F (median: 107 days; range: 6–2,275) compared to d-TGA-AS (median: 20 days; range: 5–55); when excluding one outlier, the HLHS-F median waitlist time was 105 days (range: 6–153).

**Table 1:** Demographics and clinical characteristics of nine patients with congenital heart disease (CHD) and systemic right ventricle physiology undergoing heart transplantation.

Patient and gender	CHD	Age (years)	Transplant indication	Days on waitlist including inactive time	Waitlist medical urgency at listing	Waitlist medical urgency at transplant	Race/ethnicity
1 (F)	HLHS-F	19	Systemic RV failure	6	Adult status 2	Adult status 2	Hispanic/Latino
2 (F)	HLHS-F	12	Systemic RV failure	153	Status 1B	Status 1B	White, Non-Hispanic
3 (M)	HLHS-F	30	Systemic RV failure	108	Adult status 4	Adult status 4	White, Non-Hispanic
4 (F)	HLHS-F	16	Systemic RV failure	2,275	Status 2	Status 1B	Black, Non-Hispanic
5 (M)	HLHS-F	28	Systemic RV failure	22	Adult status 2	Adult status 2	White, Non-Hispanic
6 (F)	HLHS-F	32	Systemic RV failure	105	Adult status 4	Adult status 4	White, Non-Hispanic
7 (M)	d-TGA-AS	44	Systemic RV failure	55	Adult status 4	Adult status 3	White, Non-Hispanic
8 (M)	d-TGA-AS	42	Systemic RV failure	20	Adult status 4	Adult status 4	White, Non-Hispanic
9 (F)	d-TGA-AS	38	Systemic RV failure	5	Adult status 2	Adult status 2	Hispanic/Latino

**Table 2:** Demographic and clinical summary of patients with HLHS-F or d-TGA-aS.

Variable	HLHS-F (*n* = 6)	d-TGA-AS (*n* = 3)
Recipient age (years)	24 (12–32)	42 (38–44)
Females, *n* (%)	4 (67)	1 (33)
Waitlist time (days)	107 (6–2,275)	20 (5–55)
Waitlist time (days)^a^ (outlier excluded)	105 (6–153)	20 (5–55)

^a^ One HLHS-F patient waited 2,275 days; values are reported both with and without this outlier for transparency.

### 3.2 Fibrosis

**Figure 1:**
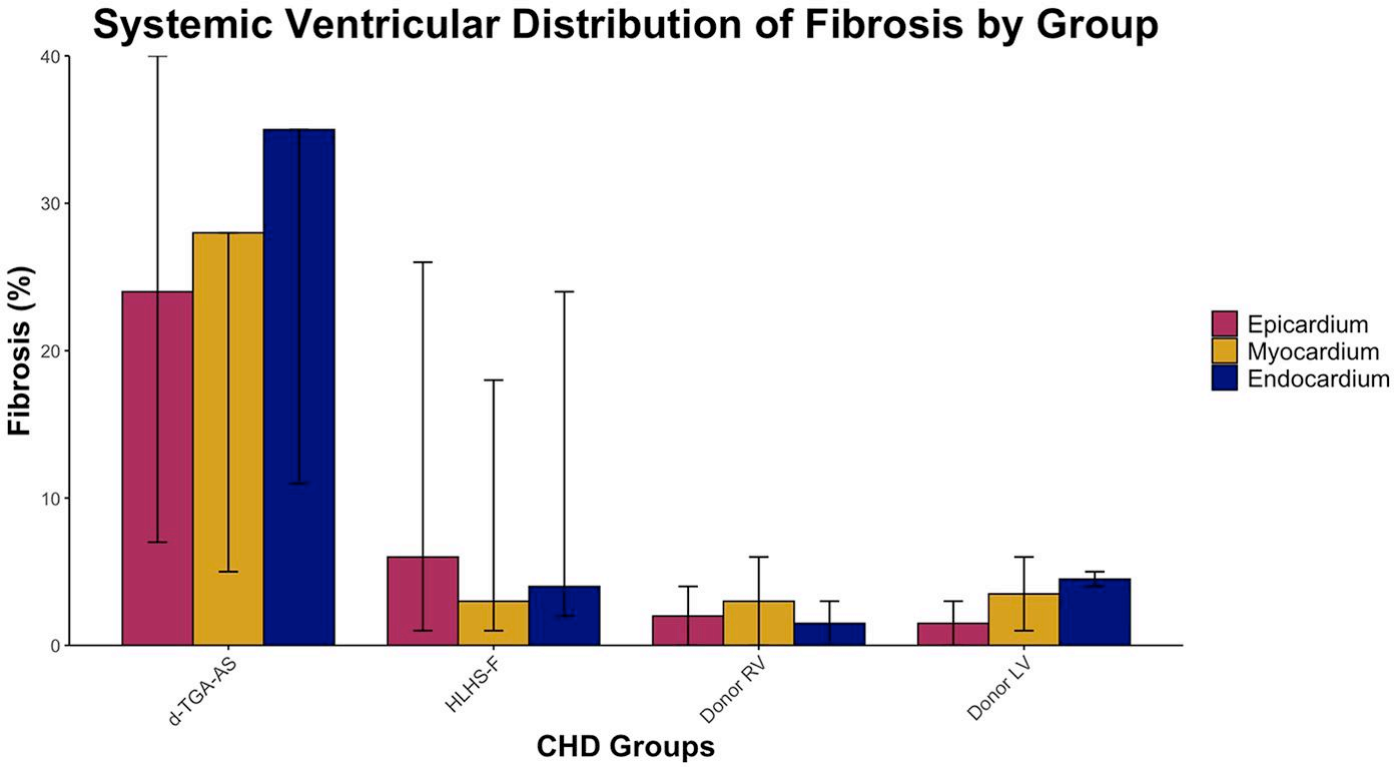
Systemic ventricular distribution of fibrosis by group. Median percentage fibrosis across epicardium, myocardium, and endocardium in HLHS-F (*n* = 6), d-TGA-AS (*n* = 3), and donor controls (RV *n* = 2, LV *n* = 2). Bars represent group median for each layer, while vertical lines show the observed range across patients.

**Figure 2:**
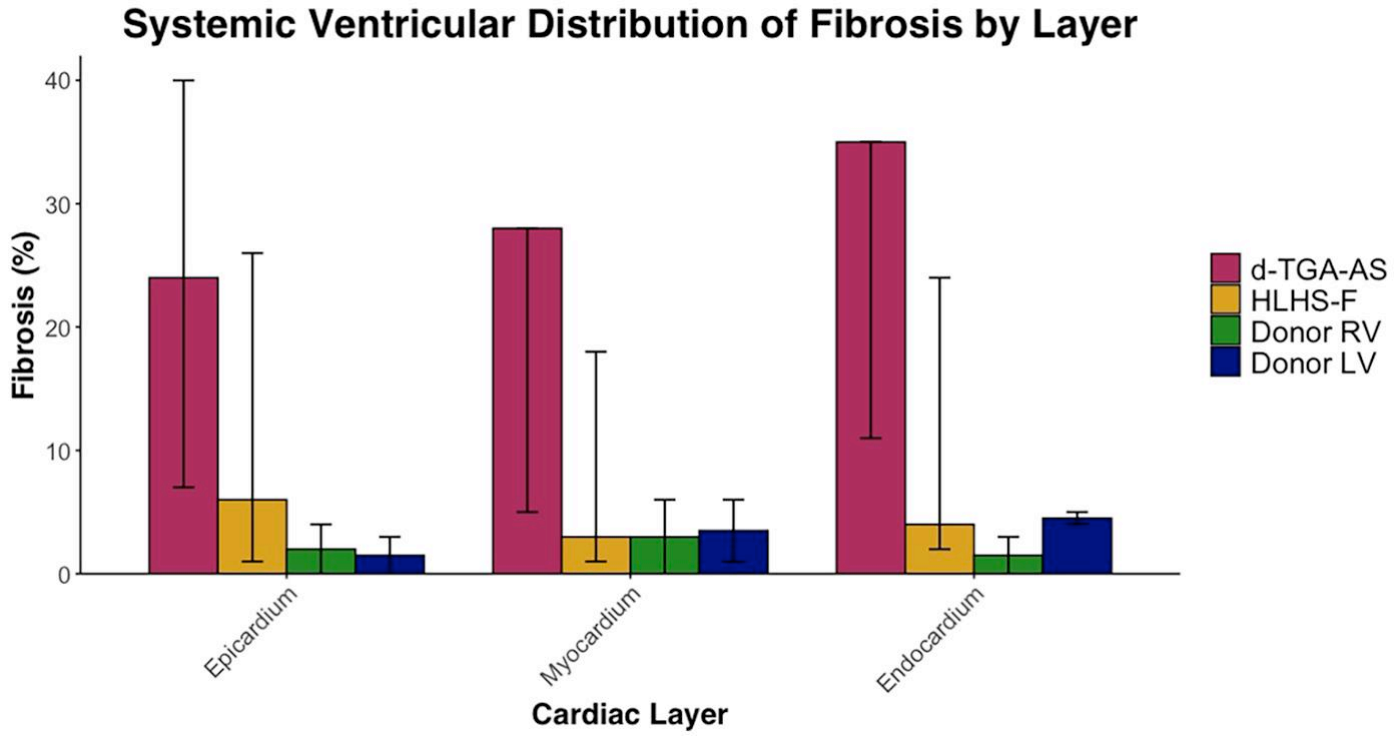
Systemic ventricular distribution of fibrosis by layer. Median percentage fibrosis in epicardium, myocardium, and endocardium in HLHS-F (*n* = 6), d-TGA-AS (*n* = 3), and donor controls (RV *n* = 2, LV *n* = 2). Bars represent group medians for each group within each cardiac layer, while vertical lines show the observed range across patients.

Fibrosis data for each patient is listed in Table 3 and summarized in Table 4. Systemic ventricular fibrosis among the CHD and control groups was highest for d-TGA-AS (Figure 1). When analyzed across the three muscle layers, the distribution of fibrosis was similar for all groups (Figure 2). In comparing fibrosis between the CHD groups, the d-TGA-AS RV showed greater fibrosis (median: 28%; range: 7–35) compared to HLHS-F (median: 4%; range: 2–24) (Table 4). RV fibrosis in both CHD groups was greater than that of the control RV (median: 2%; range: 0–4) (Table 4). LV fibrosis values were uniformly low across all groups, with medians ranging from 2% to 5% (Table 4).

**Table 3:** Per-patient systemic right ventricular fibrosis and wall thickness in patients with HLHS-F, d-TGA-aS, and donor controls.

Patient	CHD	Fibrosis (%)	Thickness (µm)
1	HLHS-F	24	11,960
2	HLHS-F	2	11,002
3	HLHS-F	4	15,745
4	HLHS-F	4	15,360
5	HLHS-F	2	12,646
6	HLHS-F	19	9,976
7	d-TGA-AS	35	8,316
8	d-TGA-AS	7	10,322
9	d-TGA-AS	28	9,063
10	Donor	0	2,582
11	Donor	4	13,386

**Table 4:** Summary of ventricular fibrosis and wall thickness in congenital heart disease transplant patients and donor controls.

Tissue	Fibrosis (%)	Thickness (µm)
RV of HLHS-F patients	4 (2–24)	12,303 (9,976–15,745)
LV of HLHS-F patients	5 (4–7)	13,882 (6,194–21,190)
RV of d-TGA-AS patients	28 (7–35)	9,063 (8,316–10,322)
LV of d-TGA-AS patients	2 (0–10)	9,886 (5,435–12,201)
RV of donor tissue	2 (0–4)	7,984 (2,582–13,386)
LV of donor tissue	3 (1–5)	17,056 (16,688–17,423)

Values are presented as median (range) for the RV and LV.

### 3.3 Thickness

Thickness data for each patient is listed in Table 3 and summarized in Table 4. Ventricular wall thickness measured at the RV mid-cavity free wall was greater in HLHS-F (median: 12,303 µm; range: 9,976–15,745) compared to d-TGA-AS (median: 9,063 µm; range: 8,316–10,322) (Table 4). Both groups had thicker RV walls than donor controls (median: 7,984 µm; range: 2,582–13,386). The donor LV mid-free wall was thickest overall (median: 17,056 µm; range: 16,688–17,423), exceeding all RV groups (Table 4).

## 4 Discussion

Heart transplant remains the gold-standard for end stage heart failure. However, according to UNOS data, the 5-year survival for heart transplantation in recipients less than fifty years old during the period from 2008 to 2011 ranged from 72.1 to 84.1% depending on age (8). Therefore, more improvements are needed in the overall management of patients with heart failure. In a circulation with a systemic RV, the mechanism for RV failure is less understood compared to left ventricular failure in the normal heart. There is a paucity of published human research that examines how the systemic RV in CHD patients fails across different structural heart configurations and physiologic conditions. The current study aimed to address this gap by characterizing the systemic RV in both single ventricular and biventricular circulations in patients at the time of transplant for ventricle failure.

**Figure 3:**
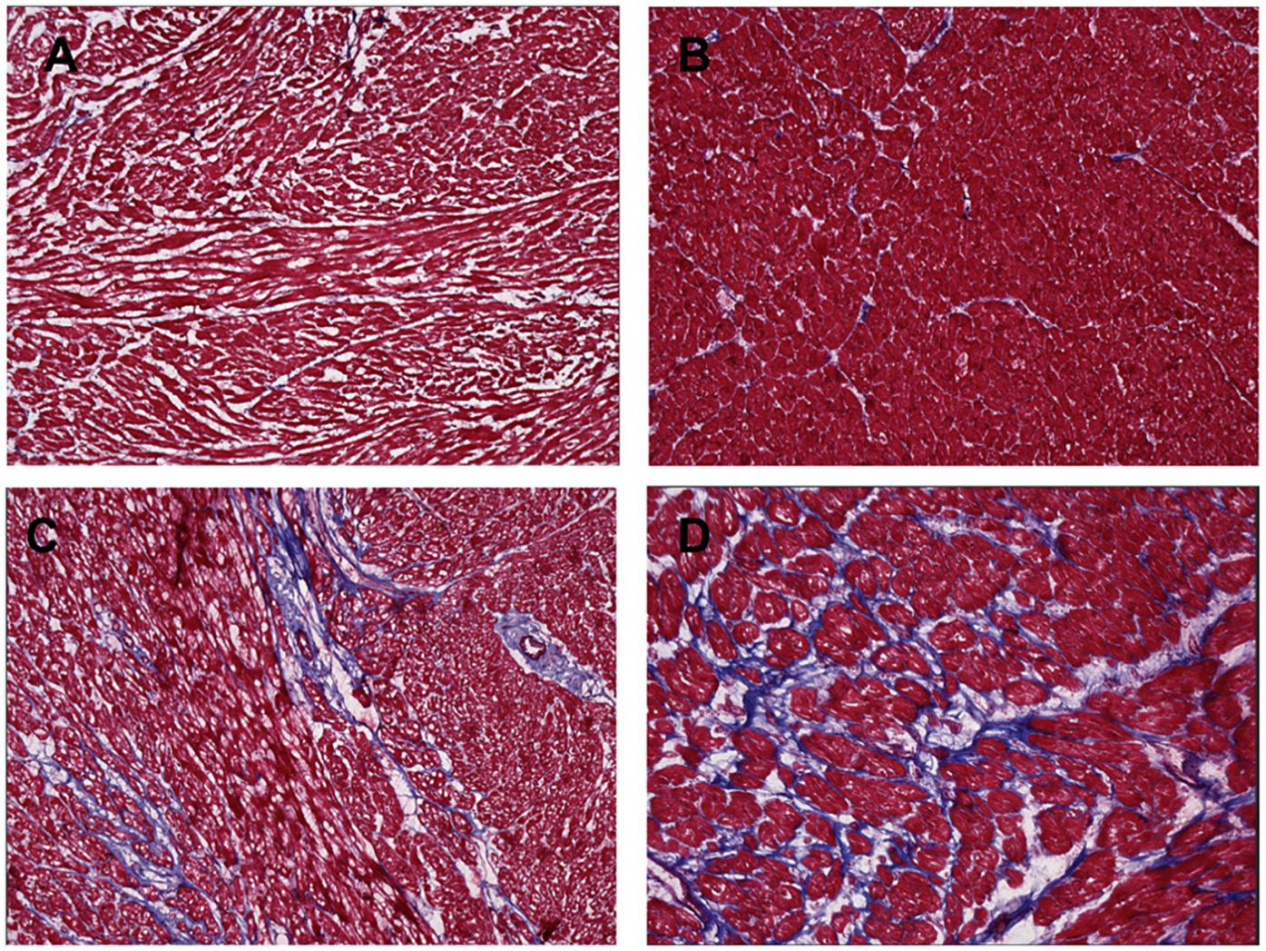
Masson's stain of ventricle tissue for donor controls and congenital heart disease (CHD) patients. Tissue was obtained from explanted hearts at time of transplant for 6 hypoplastic left heart syndrome with Fontan palliation (HLHS-F) patients, 3 and dextro-transposition of the great arteries patients with atrial switch (d-TGA-AS) patients, and 2 donor controls. Blue indicates collagen fibers and fibrosis in the ventricle tissue. **(A)** Right ventricle tissue from donor control **(B)** left ventricle tissue from donor control **(C)** systemic right ventricle tissue from HLHS-F patient **(D)** systemic right ventricle tissue from d-TGA-AS patient.

**Figure 4:**
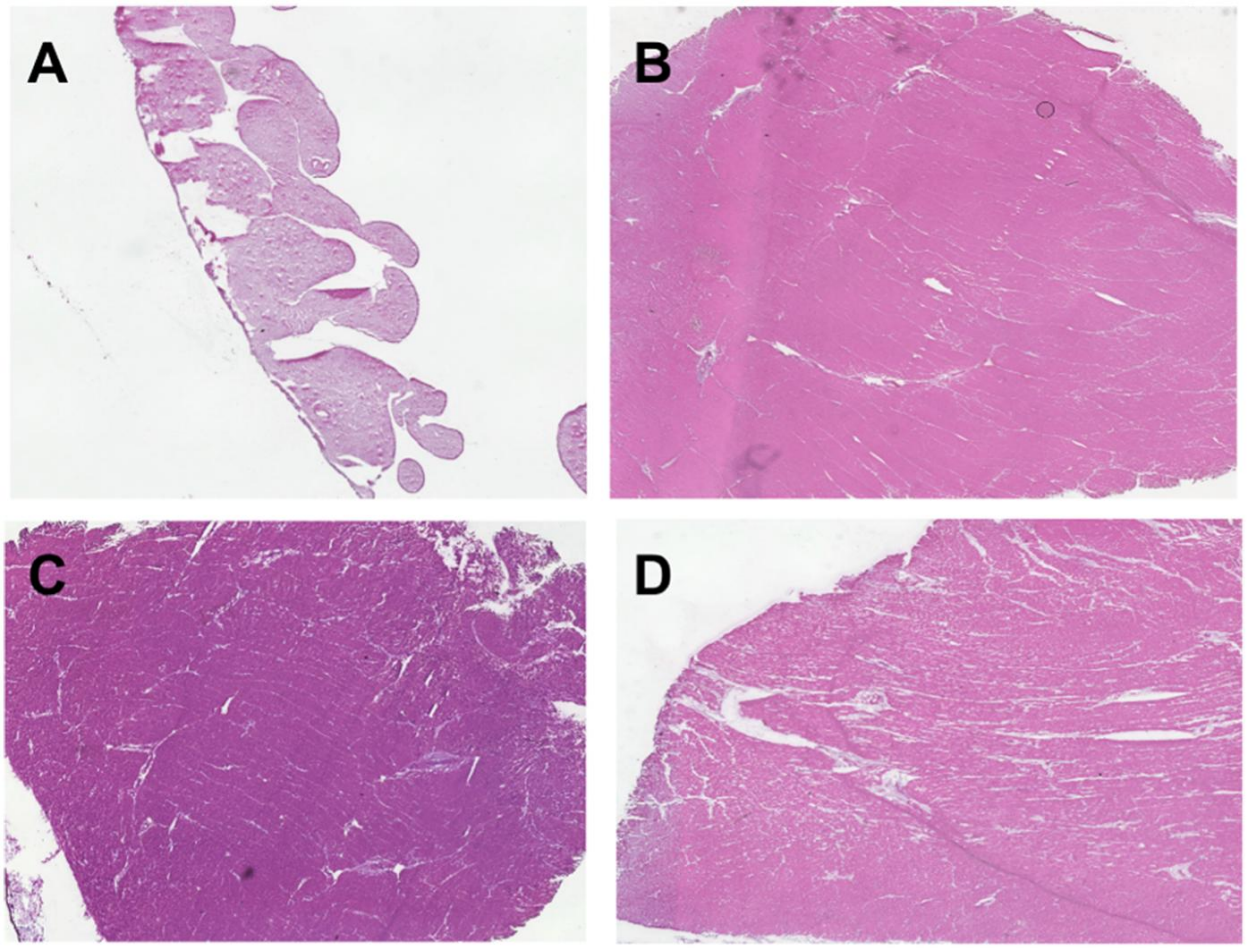
H&E staining of ventricle tissue in congenital heart disease (CHD) patients and donor controls to visualize cell architecture and ventricle thickness. Tissue was obtained from explanted hearts at time of transplant for 6 hypoplastic left heart syndrome with Fontan palliation (HLHS-F) patients, 3 dextro-transposition of the great arteries patients with atrial switch (d-TGA-AS), and 2 donor controls. **(A)** Right ventricle tissue from donor control **(B)** left ventricle tissue from donor control **(C)** systemic right ventricle tissue from HLHS-F patient **(D)** Systemic right ventricle tissue from d-TGA-AS patient.

In our approach, we analyzed nine representative images of each full-thickness RV or LV tissue sample. Fibrosis was uniform across endocardium, myocardium, and epicardium for both experimental and control specimens. The right ventricle of the CHD patients with systemic RV had higher levels of fibrosis and more hypertrophy than the control RV tissue (Figures 3,4). Potential contributors to RV failure—fibrosis and thickness—differed between d-TGA-AS and HLHSF groups. In our study, we found that patients with d-TGA-AS had higher levels of fibrosis than those with HLHS-F. This finding corroborates previous reports with imaging techniques showing greater RV fibrosis levels after atrial switch surgeries (8–11). Interstitial myocardial fibrosis has been shown to be present in newborn TGA hearts without surgical correction, with severity increasing in older uncorrected TGA patients and in TGA patients post Mustard/Senning procedure (12).Single right ventricle ischemia has also been associated with increased focal myocardial fibrosis (13). Since fibrosis is linked to increased risk for poor outcomes and likely contributes to RV failure, this may be a therapeutic target (14–16). Our histological quantification of RV fibrosis at the time of transplant adds to the current understanding of systemic RV failure and may help focus future investigations. Additionally, perfusion abnormalities consistent with ischemia and fibrosis have been demonstrated in unoperated patients with congenitally corrected TGA (ccTGA), implicating their role as contributors to RV dysfunction (17).

Singh et al. demonstrated reduced myocardial blood flow in patients with systemic RV after the Mustard procedure when treated with adenosine (18). This finding suggests the RV muscle in our d-TGA-AS group may experience reduced blood flow when demand increases, for example during exercise. Patients with complete transposition of the great arteries following the Mustard procedure show evidence of intramural coronary remodeling, with thick walls and narrowed lumens, making the RV vulnerable to ischemia (19). Capillary density is also diminished in the setting of RV hypertrophy secondary to a systemic pressure load (20). Together, these vascular abnormalities may create a supply and demand blood flow imbalance in the systemic RV such that myocardial ischemia could lead to systolic and diastolic failure. Hauser and colleagues identified clinical correlates to this explanation for RV failure in ccTGA; however, a similar relationship for d-TGA-AS was not evident (20).

Over many years following an atrial switch operation, these repetitive bouts of relative ischemia may cause myocardial injury, induce fibrosis, and eventually lead to heart failure. Hornung et al. reported that excessive RV hypertrophy in d-TGA patients following the Mustard procedure was associated with reduced systemic RV ejection fraction, likely through ischemia. In our study, the presence of high levels of fibrosis in the d-TGA-AS population at the time of transplant may represent the consequence of chronic hypertrophy-driven ischemia that developed over a longer time course, even in cases where RV thickness did not exceed controls (21). Further, our three patients with d-TGA-AS presented for transplantation at 38, 42, and 44 years respectively compared to a median age of 27 years in Hornung's cohort. It is therefore possible that the additional several years of a pressure load on the systemic RV in our d-TGA-AS group caused more fibrosis with comparatively less muscle mass.

In both HLHS-F and d-TGA-AS patients, the systemic RV pumps to the aorta against a higher vascular resistance than the native sub-pulmonary RV. However, in the d-TGA-AS group, the combination of the high systemic vascular resistance (SVR) with periodic restricted myocardial blood flow may activate fibrotic pathways and ultimately cause systemic RV failure over time. In contrast, for patients in the HLHS-F group, the obligatory passive pulmonary blood flow leads to chronic underfilling of the RV and reduced cardiac output (22–24). In combination with a relatively high SVR, these factors may induce hypertrophy and much less fibrosis initially. Over a period of years, the coupling of high SVR and chronic underfilling may accelerate ventricular myocardial failure. Rickers et al. also observed reduced myocardial blood flow in HLHS-F patients, suggesting that ischemia may contribute to this group as well (25). The time course for these two proposed processes may be substantially different and could explain the significant difference in age at presentation for transplant seen in our two study groups. Additionally, the RV hypertrophy for the HLHS-F and d-TGA-AS groups was less than the control LV, suggesting that the hypertrophic response by the RV exposed to SVR may be different than for the normal left ventricle.

Specific to HLHS, the initial volume load from parallel circulation occurs from birth and with successful staged palliation often decreases within a few months. However, there are circumstances such as atrioventricular valve regurgitation, formation of aortopulmonary collaterals, and neoaortic regurgitation that could develop over time and produce an additional volume load. Concurrently, the systemic RV pressure load remains throughout life and may worsen over time, for example with recurrent arch obstruction. There is an ever-changing landscape for the pressure and volume burden on the single RV that may promote single ventricle failure and the histologic correlates in the myocardium.

## 5 Limitations

Limitations to this study include the low total number of samples (*n* = 9), and low d-TGA atrial switch samples (*n* = 3). Therefore, the analysis is descriptive because meaningful statistical comparisons were not possible. Our study was based on single mid-free wall RV samples, which may not have fully captured the variability of fibrosis, both focal and diffuse, across the entire RV. Future studies with broader sampling may provide a more comprehensive understanding of the fibrosis pattern. Additionally, the d-TGA atrial switch population was significantly older than the HLHS Fontan population and could not be age matched. The age at time of palliation predates the electronic health record and thus clinical data could not be collected to corroborate findings. Finally, the inherent physiological differences between single and biventricular circulations limit the scope of inference in this study.

## 6 Conclusion

The primary histologic finding for the d-TGA-AS group was myocardial fibrosis, while the HLHS Fontan group showed more hypertrophy than fibrosis. It is possible that fibrosis is a time-dependent finding in an RV exposed to systemic pressure and intermittent impaired myocardial blood flow. Perhaps early surveillance for fibrosis could identify patients before heart failure develops and provide an opportunity for intervention. For patients with HLHS-F circulation, the pressure and volume loads on the RV may lead to RV failure over time and warrant close observation throughout life.

## Data Availability

The original contributions presented in the study are included in the article/Supplementary Material, further inquiries can be directed to the corresponding author.
